# The C-shaped root canal systems in mandibular second molars in an Emirati population

**DOI:** 10.1038/s41598-021-03329-1

**Published:** 2021-12-13

**Authors:** Summayah Khawaja, Nouf Alharbi, Jahanzeb Chaudhry, Amar Hasan Khamis, Rashid El Abed, Ahmed Ghoneima, Mohamed Jamal

**Affiliations:** 1 Hamdan Bin Mohammed College of Dental Medicine, Mohammed Bin Rashid University of Medicine and Health Sciences, Building 14, Dubai Health Care City, P.O.Box: 505055, Dubai, United Arab Emirates; 2 Department of Dentistry, Healthpoint Hospital, (Mubadala Health), Zayed Sports City, Abu Dhabi, United Arab Emirates

**Keywords:** Oral anatomy, Dentistry, Dental radiology, Endodontics

## Abstract

Our study aimed to describe the root and canal morphology of mandibular second molars in Emirati population and to study the prevalence and types of morphological change in C-shaped canal configuration along the root length in an effort to describe C-shaped molars as a unit. Cone beam computed tomography (CBCT) scans of Emirati patients were analyzed in multiple plans and root and canal configuration of mandibular second molars were evaluated. Moreover, specific types of morphological change in C-shaped canal configuration along root length were studied and reconstructed using 3D reconstruction software. A total of 508 mandibular second molars were evaluated. Among the non-C-shaped mandibular second molars, two separate roots were the most prevalent root morphology (78.3%). The mesial root's most common root canal configuration was Vertucci Type II (46.5%), and in the distal root, Vertucci Type I (90.5%). The prevalence of C-shaped mandibular second molars was 17.9% and was significantly prevalent (P < 0.001) in females. Specific types of morphological change in C-shaped molars along the root length were observed and described for the first time, in which the most common types of morphological change were C1-C2-C3d (18%), C1-C3c-C3d (15.4%), C4-C3c-C3d (7.7%), and C3c-C3c-C3d (7.7%). This study showed wide variations in the root and canal morphology in mandibular second molars in Emirati population with a relatively high prevalence of C-shaped canal configuration (17.9%). Moreover, specific types of morphological change in C-shaped configuration were detected and described for the first time in this population.

## Introduction

Several studies have reported that incomplete debridement of the root canal system might lead to endodontic therapy failure, due to potential presence of necrotic pulp remnants and microorganisms^1, 2^. Therefore, recognizing, locating and treating all root canal anatomy is essential to ensure successful outcome of endodontic therapy. Root and canal morphology of mandibular second molar exhibits complex anatomical variations such as C-shaped configuraiton^2, 3^. This specific morphology is characterized by presence of one to three canals that are mostly connected by thin ribbon-like or fan- shaped communications. These characteristics make complete debridement, shaping and obturation of such C-shaped configuration challenging. In addition, C-shaped molars are more prone to iatrogenic errors such as strip perforations due to the thin layer of dentin between the canal and external root wall^2^. Moreover, studies have reported the influence of ethnicity and race on the prevalence and complexity of C-shaped configuration^4, 5^. Therefore, several investigators have studied the root and canal morphology of mandibular second molar and the prevalence of C-shaped canal configuration in different populations^4, 5^. The prevalence of C-shaped mandibular molars is comparatively very high (29–45%) in East Asian populations in comparison to African, North American, West Asians and European populations (3.5–22.4%)^4, 6, 7^.

Over the years, researchers have used several methods such as staining and clearing, sectioning, conventional and digital radiographs, computed tomography and micro- computed tomography to study the root canal morphology of the teeth^2, 3^. In the recent years, use of come beam computed tomography (CBCT) has gained popularity due to its non-invasive nature, wide availability and its relative accuracy in detecting fine canal anatomy in comparison to canal staining and clearing technique^8–10^. Additionally, data generated through CBCT can be processed using software that allow three dimensional (3D) rendering and advanced analysis.

After extensive review of literature, no study has been conducted to analyze the root and canal morphology and prevalence of C-shaped configuration in mandibular second molars in Emirati population. Such information will aid the clinicians treating this population to plan their treatment and using modified techniques to manage potential anatomical complexities. Moreover, most of studies conducted so far have described and reported the C-shaped configuration at specific axial cross-sections of the root using Fan et al. classification^11^. However, a change in the configuration of C-shape canal along the length of the root has been reported^11^. Hence, there’s a lack of linking these cross-sections together to give an overall picture of the C-shaped canal anatomy from coronal to apical direction as a single unit.

Therefore, the aim of this study was to describe the root and canal morphology of mandibular second molars in Emirati population using CBCT. In addition, we aimed to study the types of morphological change in C-shaped configuration along the root length in an effort to describe the C-shaped molars as a single unit.

## Methods

This study was approved by the Research and Ethics Committees of the Mohammed bin Rashid University of Medicine and Health Sciences (Dubai, UAE) and of Healthpoint Hospital (Mubadala Health, Abu Dhabi, UAE) (Protocol no. REC0009). All study procedures were performed in accordance with the relevant guidelines and regulations of the above-mentioned institutes. For this type of retrospective study, and as recommended by the Research and Ethics Committees of the above-mentioned institutes, specific consent form is not required. Furthermore, all patients or their legal guardians that are treated at Healthpoint Hospital provide written informed consent for the use of their records for research purposes.

### Sample collection

CBCT scans of patients who were treated at Healthpoint Hospital (HPH), department of dentistry between 2017 and 2018 were obtained and analyzed. The CBCT scans were acquired using the Orthophos SL (Dentsply Sirona, USA). The imaging protocol was as follows: Field of view (FOV) = 8 × 8 cm; tube peak potential = 85kVp; tube current = 7 mA; time = 5 s; voxel size = 0.15 mm. CBCT scans of patients who met the following criteria were included in the study: (i) Emirati population (holding United Arab Emirates Citizenship), (ii) Age range 15–75 years (iii) presence of bilateral mandibular permanent second molars (iv) Fully matured and erupted teeth. Molars with root canal fillings, posts, crowns, coronal or root resorption, extensive coronal and root caries and periapical or periradicular radiolucency were excluded.

The sample size (254 CBCT scans) was determined based on Cochran’s sample size, where prevalence of C-shaped mandibular second molars in previous studies was considered the relevant difference. The CBCT scans were randomly selected and anonymized using “Anonymize” tool of Horos image processing software (Nimble Co LLC d/b/a Purview in Annapolis, MD USA) to remove all patient identifiers^12^. To ensure randomization during selection, the available scans were listed in Microsoft Excel sheet and were ordered based on the date they were obtained. These scans were given a random unique number using the random function in Microsoft Excel. Then they were reordered using the ascending function in Microsoft Excel, making a random list of the scans. Next, the principal investigator (PI) reviewed the scans starting from the beginning of the random list and the first 254 scans that fulfilled the inclusion/exclusion criteria were selected for the study. The selected scans were exported from HPH CBCT database in Digital Imaging and Communication in Medicine (DICOM) format.

### Radiographic evaluation

**Figure 1:**
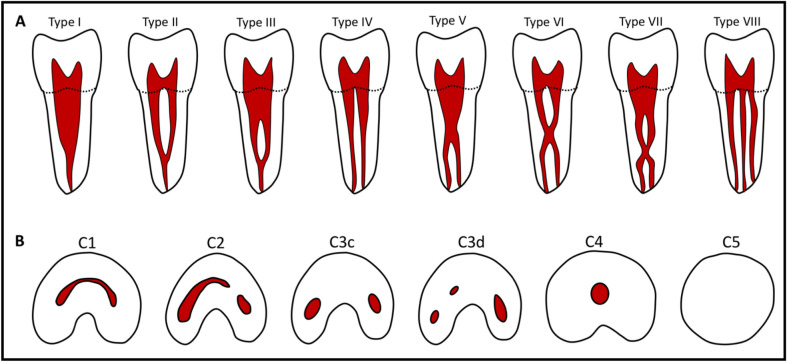
Illustration of (**A**) Vertucci classification of root canal system and (**B**) Fan et al. classification of C-shaped canal configuration.

After data extraction and anonymization, 2 evaluators (the principle investigator, and an expert endodontist) evaluated all scans on an iMAC computer (27-inch screen size with Retina 5 K display, 5120 × 2880 resolution with support for 1 billion colors, 500 units brightness) in a room with controlled lighting using Horos DICOM viewer^12^. The evaluators assessed the canal and root morphology using a 3D multiplanar reconstruction tool (3D MPR), in which all images were examined in the axial, coronal, and sagittal plans. Furthermore, the observers determined the coronal section to be within 2 mm of cemento-enamel junction (CEJ), middle third section to be within 2 mm of mid root length (from apex to CEJ) and apical section to be at apex and 2 mm above. Mandibular second molars were categorized as non-C-shaped and C-shaped based on Fan et al. criteria^11^, in which, C-shaped molar should exhibit the following features: (a) fused roots, (b) a longitudinal groove on lingual or buccal surface of the root, and (c) at least one cross-section of the canal belongs to the C1, C2, or C3 configuration (Fig. 1B).

Vertucci classification (VC)^13^ was used to assess the canal morphology of non-C-shaped mandibular molars (Fig. 1A), while for C-shaped molars, Fan et al. classification was used. (Fig. 1B). Moreover, the number of roots and symmetry of the canal morphology were recorded. Finally, the findings were tabulated and correlated with gender and tooth location.

Furthermore, for C-shaped molars, the axial cross-sectional morphology was analyzed on the CBCT to study the changes of the morphology along the length of the root and to explore the possibility of presence of specific types of morphological change in C-shaped canal configuration. After determining the most common types of change in C-shaped morphology, sample of the teeth exhibiting these morphological changes were segmented semi-automatically using 3D Slicer open-source software (http://www.slicer.org). ^14^. Each segmentation consisted of a contour of the tooth (enamel, dentin, crown and roots) and pulp system. Dolphin software (Dolphin Imaging and Management Solutions, http://www.dolphinimaging.com) was used to produce 3D video showing the changes along the root length.

To ensure reproducibility and reliability of the data, several measures were taken. First of all, before the evaluation process each evaluator was trained and calibrated using a sample of scans showing different types of root canal morphologies. Second, the scans were assessed independently, twice by both evaluators with intersession delay of 30 days. In case of disagreements, a third evaluator (endodontist) was consulted. Finally, the data was analyzed using kappa test, and the Altman’s scale was used for interpretation.

### Statistical analysis

Data was analyzed using SPSS for Windows version 25.0 (SPSS Inc., Chicago, IL). Results were cross tabulated to examine the dependency between variables. Statistical analysis was performed using χ2 (Chi-square) to determine the association between variables such as distribution of C-shaped mandibular second molar by gender and tooth location. Kruskal–Wallis test was used to determine the significance between different patterns of change. Kappa test was used to test inter- and intra-rater examiners reliability. Frequency tables’ bar graphs were used as descriptive statistics. A *P*-value of less than 0.05 was considered significant in all statistical analysis.

## Results

Overall, 1415 CBCT scans were reviewed and 254 CBCT scans were selected based on inclusion and exclusion criteria. The two evaluators reviewed the 254 CBCT scans independently (total of 508 molars) and kappa test indicated a very good intra (0.880) and inter (0.838) evaluators’ agreement based on Altman’s scale.

**Figure 2:**
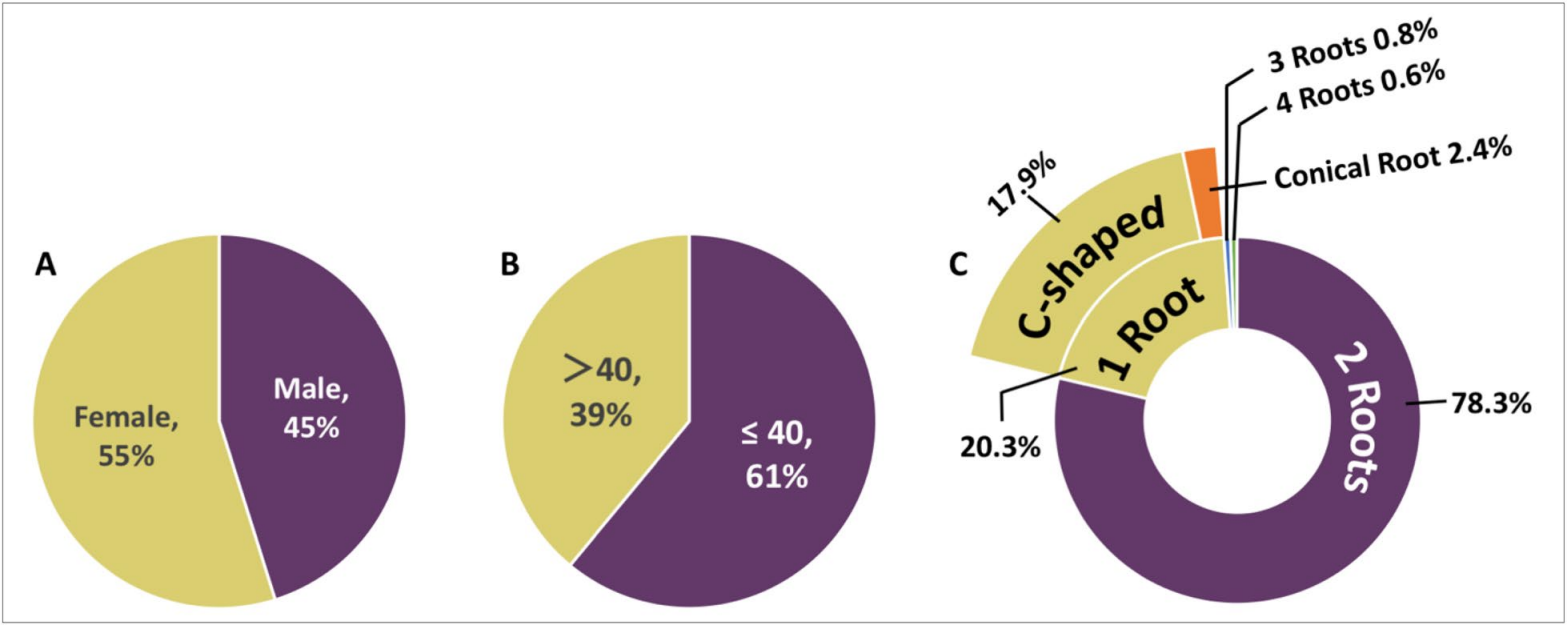
Pie charts showing (**A**) gender and (**B**) age data. (**C**) is a Pie chart indicating the percentage of number and morphology of roots of the studied mandibular second molars.

The analysis of the selected scans showed that 115 scans (45.3%) were for male patients and 139 (54.7%) for female patients with age that range from 15–75 years (Fig. 2A). Of the 254 patients, 61% were in ≤ 40 age group and 39% were in age group > 40 (Fig. 2B).

### Root morphology and number of roots

The initial analysis showed that 82.1% were non-C-shaped molars while 17.9% were C-shaped molars (Fig. 2C). Among the non-C-shaped mandibular second molars, two roots were the most common (78.3%) followed by single rooted molars (2.4%) (Fig. 2B). The prevalence of mandibular second molars with three and four roots was very low (0.8% and 0.6%, respectively) (Fig. 2B). In the molars with three roots, the third root was in the form of a mesio-lingual root (50%), mid-buccal root (25%) and mid lingual root (25%).

### Canal morphology of non-C-shaped molars

**Figure 3:**
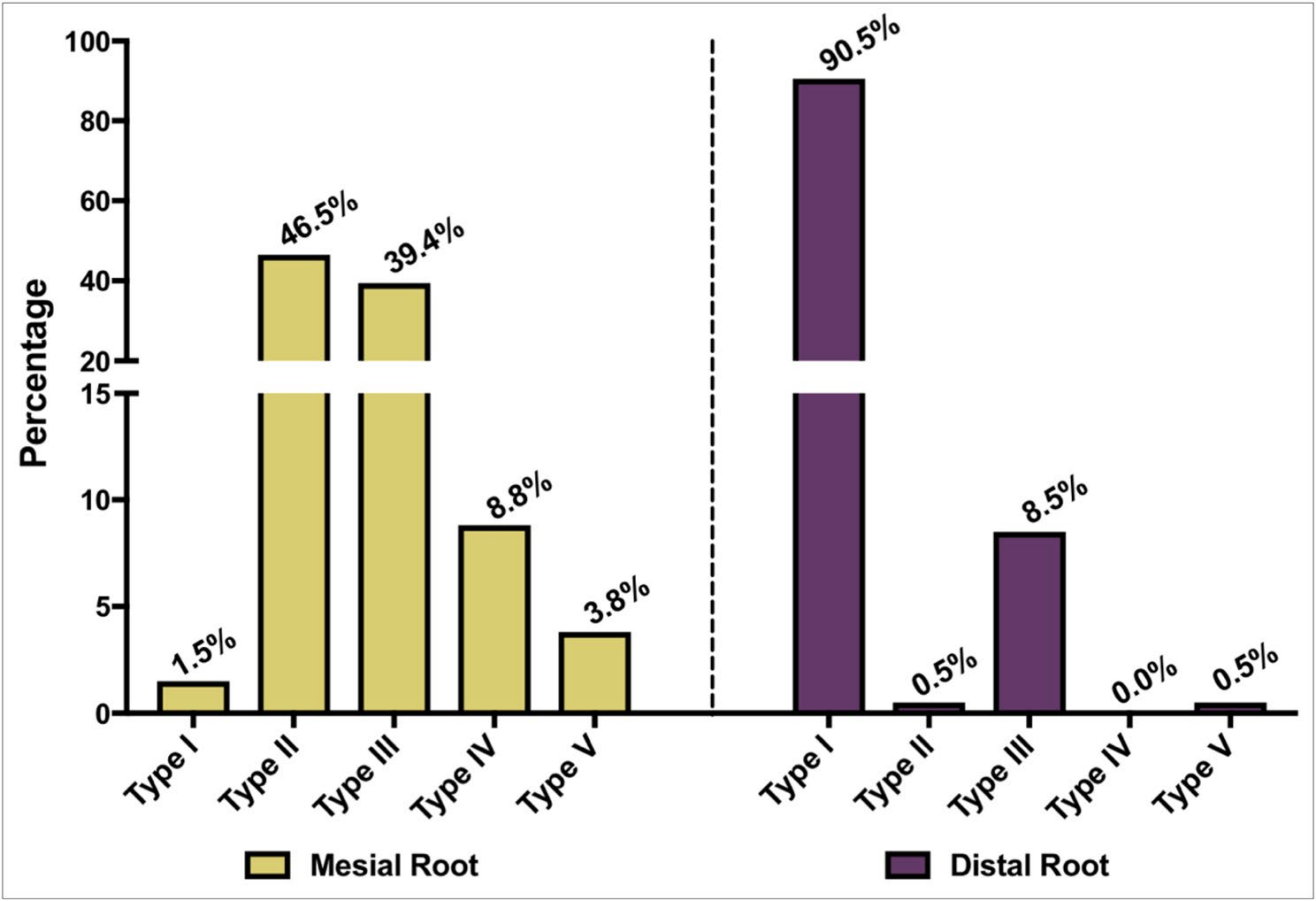
Bar chart indicating the percentages of different types of VC in the mesial and distal roots in the two rooted non-C-Shaped mandibular second molars.

There were wide variations in the canal configuration of mesial roots of two-rooted mandibular second molars (Fig. 3). The most prevalent canal configuration in the mesial roots of two-rooted molars were Type II (46.5%) and Type III (39.4%) VC (Fig. 3). The least commonly observed canal configurations were Type I (1.5%), Type V (3.8%) and Type IV (8.8%) VC (Fig. 3). In contrast, 90.5% of the distal root had Type I canal configuration. Other least common types found in the distal root were Type III (8.5%), Type II (0.5%) and Type V (0.5%) (Fig. 3). Type VI, VII and VIII VC were not found in both roots. All the mandibular second molars with conical roots, had Type I VC.

### Canal morphology of C-shaped molars

**Figure 4:**
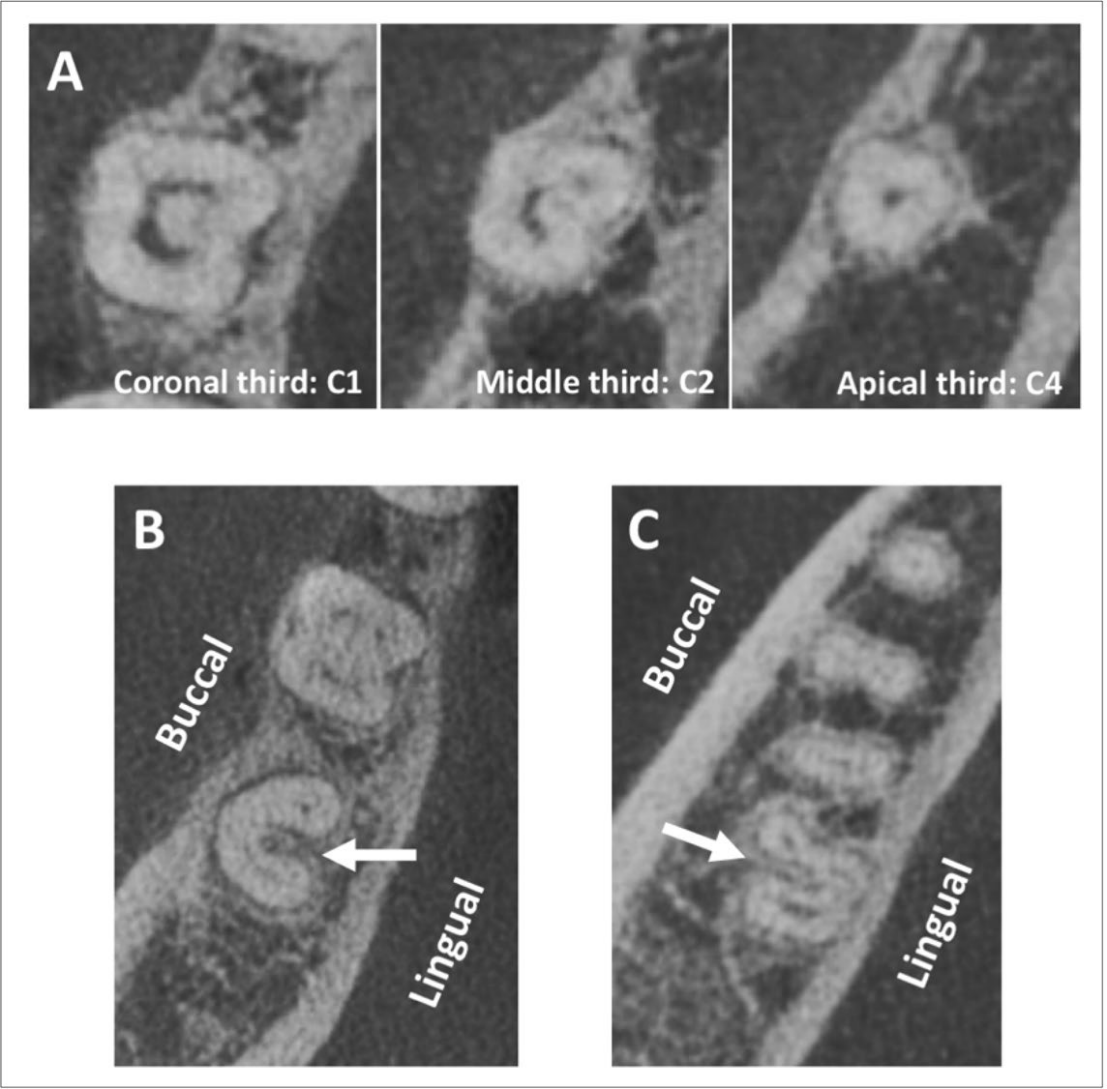
(**A**) axial cross section of mandibular molar showing the changes in the C-Shaped configuration along the root length; at the coronal third the canal started as C1, then changed into C2 at the middle third and finally ended as C4 at the apical third. (**B**) axial cross section of a mandibular second molar showing a lingual location of the groove, while (**C**) is showing the buccal location of the groove.

The canal configuration of C-shaped molars was classified using Fan et al. classification (12). The most common canal configuration at the coronal level was C1 (41.75%), while C3c (51.7%) and C3d (65.9%) were most common at the middle and apical thirds, respectively. Of the 91 C-shaped mandibular second molars, only 5.5% showed no changes in the C-shaped canal along the length of the root. Whereas, in 94.5% molars the canal configuration changed from the coronal to apical direction (Fig. 4A, Supplement 1).

Interestingly, in the present study, 4 out of 5 C-shaped mandibular molars with unchanged canal morphology had C3d canal configuration and one C-shaped molar had C2 canal configuration throughout the root. Of the 91 C-shaped mandibular second molars, only 11 molars (12%) had groove on the buccal surface of the roots (Fig. 4C), whereas 80 molars (88%) had groove on the lingual surface (Fig. 4B).

**Figure 5:**
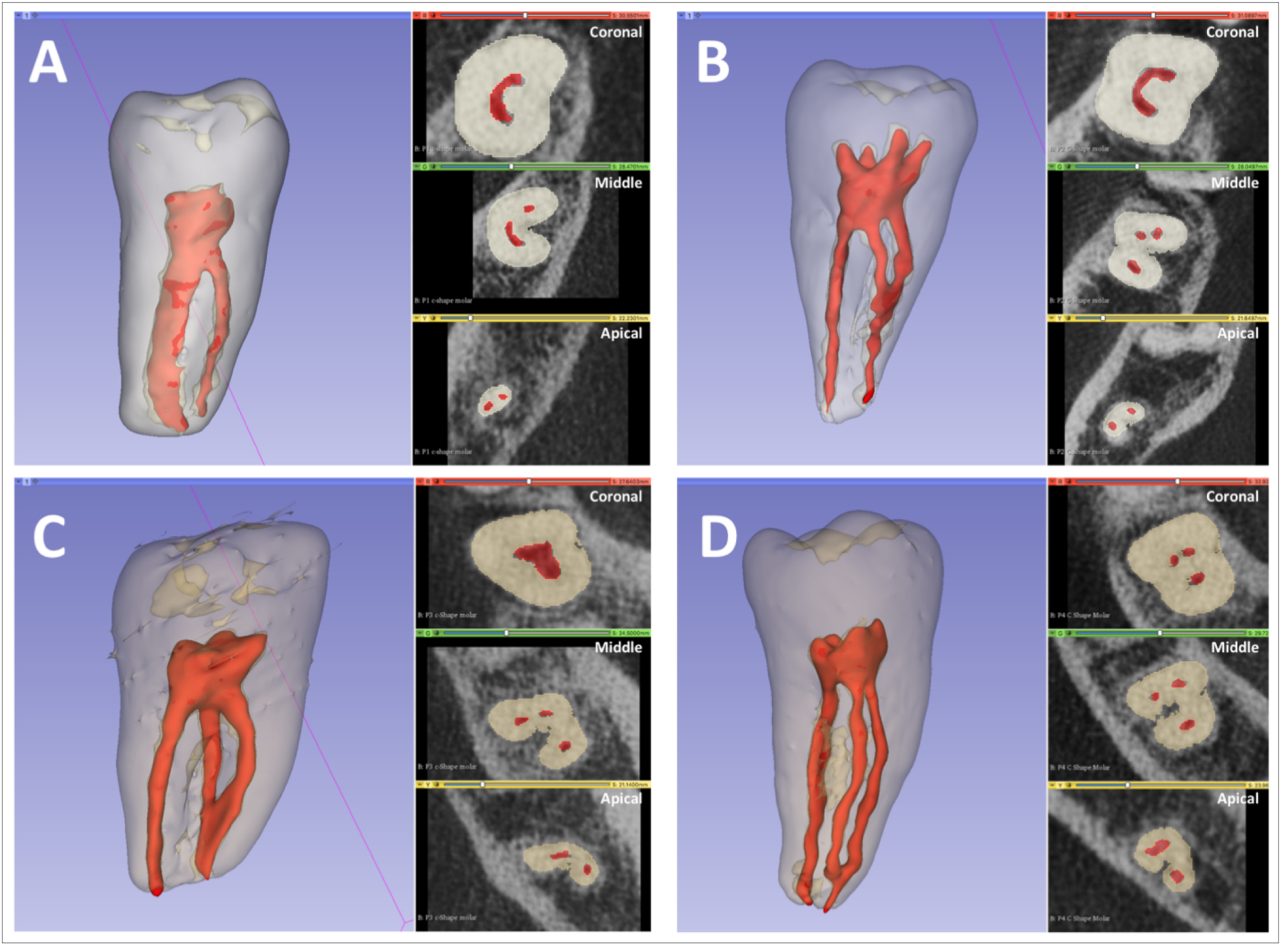
3D reconstruction of the most common types of morphological change in C-shape configuration along the root length; (**A**) T1: C1-C2-C3d, (**B**) T2: C1-C3c-C3d, (**C**) T3: C4-C3c-C3d and (**D**) T4: C3c-C3c-C3d.

The analysis of C-shaped mandibular second molars with changes in canal configuration along the length of the root showed that there were 18 types of morphological change in which 4 of them (48.8%) were more significantly present (p < 0.001). More specifically, in the most common type (T1), the canal started as C1 then changed into C2 at the middle third and ended as C3d at the apical third (C1-C2-C3d), in 18% of the C-shaped mandibular second molars (Table 1). The other common types of morphological change were T2: C1-C3c-C3d (15.4%), T3: C4-C3c-C3d (7.7%) and T4: C3c-C3c-C3d (7.7%) (Table 1, Fig. 5). Figure 5 illustrate 3D reconstructions of most common types of morphological change in C-shaped mandibular second molars. The rest of the 14 types of morphological change (51.2%) were insignificantly present.

**Table 1:** Types of morphological change in C-shape canal configuration along the root length.

Types of morphological change	Root thirds			Total percentage
	Coronal	Middle	Apical	
Type 1 (T1)	C1	C2	C3d	18%
Type 2 (T2)	C1	C3c	C3d	15.4%
Type 3 (T3)	C4	C3c	C3d	7.7%
Type 4 (T4)	C3c	C3c	C3d	7.7%

### Prevalence of C-shaped mandibular second molars according to gender and tooth location

On testing the association between the prevalence of C-shaped mandibular second molar and gender, C-shaped molars were significantly more prevalent in females (75.8%) than in males (24.2%) (*P* < 0.001). No significant correlation was found between the prevalence of C-shaped mandibular second molars and tooth location (right side vs left side). Around 54% were located on the right side and 46% on the left side (*P* = 0.244).

### Bilateral symmetry of C-shaped mandibular second molars

The analysis of bilateral symmetry showed that 71.7% of the C-shaped mandibular second molars were bilaterally symmetric (*P* < 0.001). In case of patients with unilateral C-shaped molars (28.3%), majority of C-shaped molars (73.3%) were located on the right side.

## Discussion

The effect of ethnicities on the root and canal morphology of mandibular second molar is well documented in the literature^4, 5, 15^. Furthermore, mandibular second molar is the most common tooth to exhibit C-shaped canal morphology^3^. Here we investigated the root and canal morphology of mandibular second molar in Emirati population. We have also described and studied the changes in the C-shape configuration along the root length. This is an effort to address the knowledge gap related to this population and an attempt to describe the C-shaped molars as a single unit.

In the present study the most common root morphology in mandibular second molars was two separate roots (78.3%), one mesial and one distal. The other types of root morphology observed were molars with single conical root (2.4%), three roots (0.8%) and four roots (0.6%). Similar findings of presence of two roots followed by 3 roots in mandibular second molars have been reported in several studies^16, 17^. The prevalence of three rooted mandibular second molar documented in other populations ranges from 0.26 to 8.98%^5, 18, 19^. The third root observed in our study was found lingually in 75% of cases, which is in agreement with reports in other population^17, 20, 21^. Therefore, despite the low prevalence, the clinicians should be aware of such morphological variations in the number of roots, to successfully manage endodontic treatment in these cases.

According to our findings, Type I VC (90.5%) was the most prevalent configuration in the distal root. However, Type II (46.5%) and III (39.4%) VC were the most common root canal configurations in the mesial root. In contrast to our findings, Type II and Type IV VC were more prevalent in the mesial root of most other studied populations^16, 18, 22, 23^. The high prevalence of type III VC in mesial root has been reported in very few studies (range 26.2 to 48.45%)^15, 24, 25^. According to our findings, Vertucci Type VI, VII and VIII were not detected in the sample of mandibular second molars examined. This is in agreement with several other published studies^18, 19^. Therefore, our results showed that Type II VC is the most common canal configuration in the mesial root in Emirati population. Thus, the clinicians treating this population should pay special attention in managing the mesial root of non-C-shaped mandibular second molar. Modifications of cleaning and shaping technique, maybe considered to prevent tearing the common apical foramen and avoid stripping, ledging and instrument fracture^26^. Moreover, the second most common canal configuration in the mesial root is Type III VC. Therefore, clinicians should explore for a second canal even if there is one orifice coronally. The inability to detect and treat the second canal might lead to endodontic failure.

**Figure 6:**
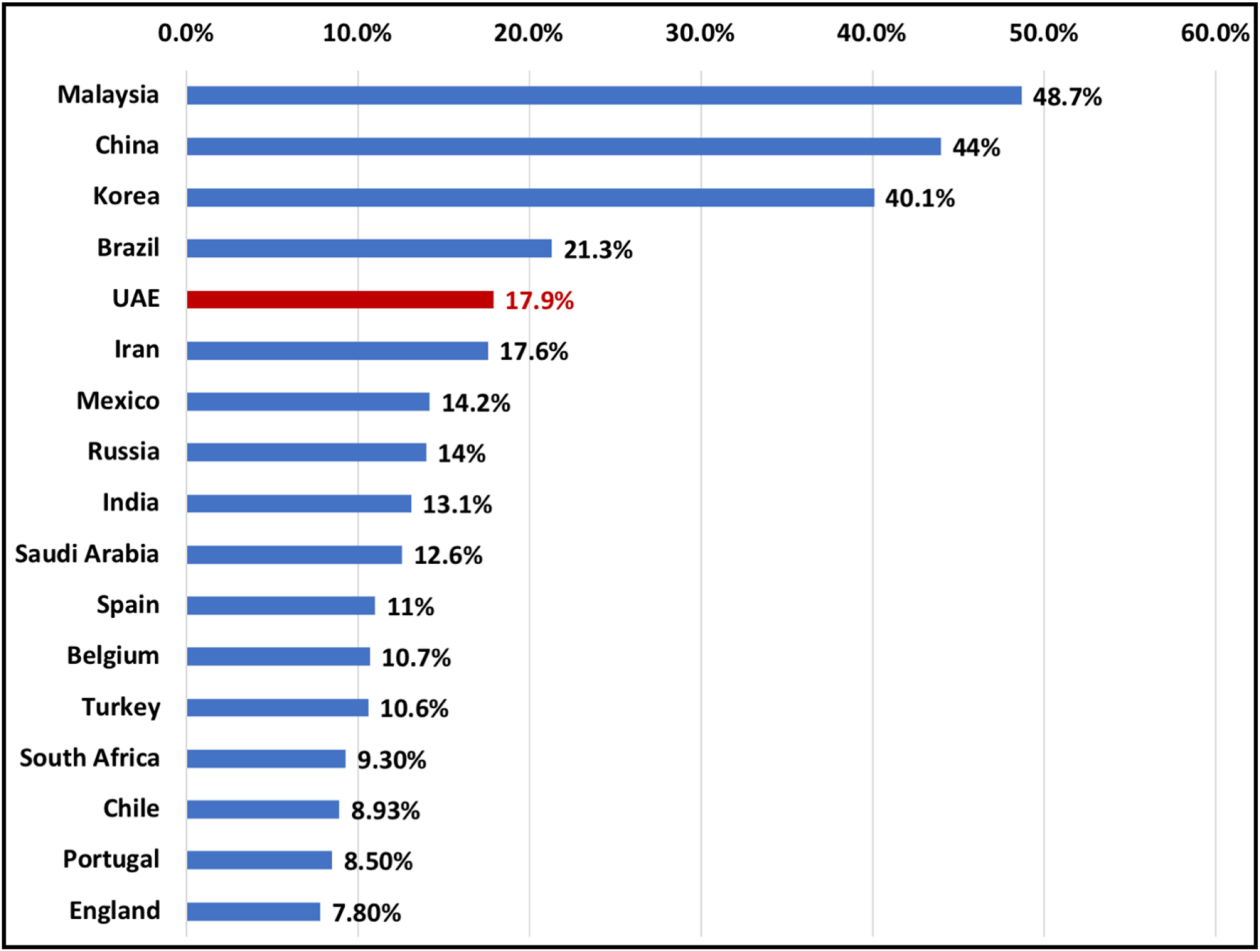
Bar chart indicating C-shaped mandibular second molar prevalence in different populations. These studies were conducted using CBCT.

There is a wide variation in prevalence of C-shaped mandibular second molars based on ethnicity and population studied. The prevalence in different populations ranged from 3 to 48.7%^5, 19^ (Fig. 6). According to our findings, the prevalence of C-shaped mandibular second molars in Emirati population was 17.9%. The prevalence is relatively higher compared to other middle eastern countries such as Saudi Arabia 7.9–12.57%^27, 28^ and Turkey 4.1–10.6%^22^ but lower than that of Chinese, Korean and Malaysian population (44–48.7%)^5, 19^. Moreover, our analysis showed that C-shaped molars were significantly higher in females compared to males (P < 0.005). Our results are in agreement with other studies which reported an association between gender and high prevalence of C-shaped molars^4, 5^.

Furthermore, the effect of ethnicity has also been reported on C-shaped canal configuration at different thirds of the root. In Iranian population, the most common canal configuration at the coronal level was C1 (50%), in the middle C3d (32.9%) and apical C3d (36.6%)^29^. Whereas, in Saudi Arabia, C3c was most common (37.1%) at coronal third, C3c (32.3%) in the middle third and C3d (30.6%) in apical third^30^. In the present study, the most common canal configuration in C-shaped mandibular molars at the coronal, middle and apical level was C1(41.75%), C3c (51.7%) and C3d (65.9%) respectively.

In the present study, the majority of the C-shaped mandibular second molars had a lingual radicular groove (88%) while only 12% had buccal radicular groove. Our findings are similar to those findings of Jin et al. and Kim et al. in Korean population^31, 32^, Martin et al. in Portuguese population^33^ and Alfawaz et al. in Saudi population^30^, in which the prevalence of buccal radicular groove was less than that of lingual radicular groove (1–22.6% prevalence of buccal radicular groove). In contrast to our findings, Ladeira et al. observed the presence of buccal radicular groove in more than two thirds (69.4%) of the C-shaped mandibular second molars in Brazilian population^34^. As documented, the dentin thickness is least at the groove area^35^. Therefore, knowing the location and direction of the groove is important to avoid overpreparation of the canal at that region which can result in iatrogenic perforation.

Moreover, we observed a change in the C-shaped canal configuration coronal to apical in 94.5% of the studied C-shaped mandibular molars. This is similar to the findings of Zheng et al. in Chinese population^6^. Whereas, the change in canal configuration was observed only in 66% of C-shaped molars in Korean population^32^. However, the types of morphological change along the length of the root of C-shaped canal configuration was not studied in those populations. Describing the changes in C-shaped canal morphology along the length of the root, can allow researchers and clinicians to have a better understanding of C-Shaped molars as a single unit. This will allow developing new treatment strategies to manage such teeth. Here, we attempted to study such a change and our analysis revealed 4 common types of morphological change in the C-shaped mandibular second molars. Specifically, the most common type was T1: C1-C2-C3d (18%) followed by T2: C1-C3c-C3d (15.4%), T3: C4-C3c-C3d (7.7%) and T4: C3c-C3c-C3d (7.7%). This finding could indicate an effect of ethnicity on presence of specific types of morphological change in C-shaped canal configuration in certain population. However, further studies in other populations are required to confirm such association. Such information if available, can help the clinicians to manage these cases, in addition to other available tools and techniques.

C-shaped canal configuration with the presence of narrow ribbon-like and fan-shaped areas, transverse anastomoses, lateral canals and apical delta make the cleaning and shaping of these teeth challenging^2^. With the relatively high prevalence in Emirati population, clinicians should consider using advanced tools to diagnose and manage such complex anatomy such as CBCT^10^, Dental operating microscope^36^, advanced irrigation activation and delivery systems (such as passive ultrasonic irrigation and negative pressure, laser activated irrigation)^37–39^ and calcium hydroxide as intra canal medicament^3^. Furthermore, as the most common types of morphological change in C-shaped molar ends up with C3d apically, clinicians should make sure to locate and clean both canals to avoid any failures.

One limitation of our study is that it’s a retrospective study, therefore the inability to control certain factors like FOV, voxel size and the quality of CBCT scan image. Therefore, in the present study, overall image resolution and quality was influenced due to the medium size FOV (8 cm × 8 cm) CBCT scans. However, the voxel size used was 0.15 which is considered acceptable when compared to other studies^40^. Furthermore, further studies are required in different population to determine the effect of ethnicity on the pattern of change in C-shaped molar along the root length. Another limitation of this retrospective study is that ethnicity was determined based on holding UAE citizenship. Therefore, the data may not represent the whole UAE population, as the UAE nationals represent almost 11% of the total population^41–43^. However, the results of this study are important for clinicians treating UAE nationals, as there is an overall agreement that there is low variation in the ethnic groups among the UAE nationals^41^.

## Conclusion

Based on our literature review, this is the first study to investigate the root and canal morphology of mandibular second molars in a sample of Emirati population. According to our findings wide variations of canal configuration is observed in the mesial root of mandibular second molars. In addition, a relatively high prevalence (17.9%) of C-shaped mandibular molars is found in Emirati subpopulation. Moreover, specific types of morphological change in the root canal system of C-shaped molars were observed and described for the first time. The results of this study emphasize on the significance of knowledge of root canal morphology beside using advanced technology in developing modified clinical approaches to successfully treat these cases.

## Supplementary Information

Supplementary Video 1.

Supplementary Information 1.
