# Supplementary material for: The C-shaped root canal systems in mandibular second molars in an Emirati population
Source: Sci Rep. 2021 Dec 13;11:23863. doi: 10.1038/s41598-021-03329-1 (PMC8668958; doi:10.1038/s41598-021-03329-1)
Supplement: Supplementary file 2 — Supplementary Information 1. [file 41598_2021_3329_MOESM2_ESM.docx]

**Supplementary Material Legends**

**Supplement 1:** Video showing a C-shaped mandibular second molar with a change in the configuration along the root length.
